# The Mediterranean, DASH, and MIND diets and the incident of hypertension over a median follow-up of 7.4 years in the Tehran Lipid and Glucose Study

**DOI:** 10.1186/s12889-022-14843-w

**Published:** 2022-12-17

**Authors:** Elham Razmpoosh, Nazanin Moslehi, Shima Abdollahi, Sepideh Soltani, Parvin Mirmiran, Fereidoun Azizi

**Affiliations:** 1 Nutrition and Endocrine Research Center, Research Institute for Endocrine Sciences, Shahid Beheshti University of Medical Sciences, Tehran, Iran; 2 Department of Nutrition, School of Public Health, North Khorasan University of Medical Sciences, Bojnurd, Iran; 3 Yazd Cardiovascular Research Center, Shahid Sadoughi University of Medical Sciences, Yazd, Iran; 4 Department of Clinical Nutrition and Dietetics, Faculty of Nutrition and Food Technology, National Nutrition and Food Technology Research Institute, Shahid Beheshti University of Medical Sciences, Tehran, Iran; 5 Endocrine Research Center, Research Institute for Endocrine Sciences, Shahid Beheshti University of Medical Sciences, Tehran, Iran

**Keywords:** Diet, Blood pressure, MIND, DASH, Mediterranean diet

## Abstract

**Background:**

Despite the favorable effects of well-known dietary patterns in the treatment of hypertension (HTN), such as the Mediterranean (MED) and Dietary Approach to Stop Hypertension (DASH) diets, it is uncertain if adherence to these diets can reduce the risk of HTN, especially in non-Mediterranean populations. Moreover, none of the previous studies evaluated the association between the MED-DASH Intervention for Neurodegenerative Delay (MIND) diet adherence and the incidence of HTN. Therefore, we aimed to assess the association of adherence to these diets with the development of HTN in adults.

**Methods:**

This prospective study included 2706 adults free of HTN who were selected from the Tehran Lipid and Glucose Study. The MED, DASH, and MIND diet scores were computed at baseline using dietary information collected with the food frequency questionnaire. Associations between the dietary indices and risk of HTN over a median follow-up of 7.4 years were examined using Cox proportional hazards regression analysis.

**Results:**

The baseline mean age of participants was 37.9 ± 12.5 years (age range: 20–79 years), and 52.4% were women. During the 18262 person-years follow-up, 599 incidents of HTN were identified. There was no significant relationship between the dietary scores and the risk of HTN, either as continuous or categorical variables, even after excluding individuals with early/late HTN diagnosis, prehypertension, diabetes, or chronic kidney disease at baseline. A significant interaction was found between body mass index (BMI) and DASH (*P*-interaction < 0.001). Stratified analyses based on baseline BMI status revealed an inverse association between DASH and HTN risk in individuals with normal-weight (HR = 0.84, 95% CI = 0.71–0.98, *P* = 0.031), although this association did not reach statistical significance across the tertiles of DASH.

**Conclusions:**

In this study, MED, DASH, and MIND showed no significant association with the occurrence of HTN in adults. Further prospective studies on diverse populations are required to assess whether adherence to the MED, DASH, and MIND diets is an effective strategy for reducing the occurrence HTN.

## Background

Hypertension (HTN), the leading risk factor for cardiovascular disease, is a major concern in public health that affects millions of individuals throughout the world [1]. According to the World Health Organization, the number of people with HTN has increased over the last 50 years, with a greater increase in low- and middle-income countries [2]. Although HTN is a multifaceted condition, primary preventive and therapeutic approaches have been focused entirely on lifestyle modification, with dietary considerations at the top of the list [3]. Meta-analyses of randomized controlled trials (RCTs) indicate that the Mediterranean (MED) and Dietary Approach to Stop Hypertension (DASH) diets reduce blood pressure compared to the usual diet [4, 5]. In addition, a meta-analysis of observational studies estimated 13% lower odds of HTN in those on higher vs. lower adherence to the MED diet. However, most of the studies included in this meta-analysis (87.5 percent) were cross-sectional, making it impossible to determine a cause-effect association [6]. Therefore, it is unclear whether adherence to the diets can decrease the HTN risk, particularly in non-Mediterranean populations. In a large-scale cross-sectional research of Iranian adults, neither the DASH nor the MED diets were linked to high blood pressure [7]. Likewise, a prospective study of 4793 Iranian adults found no significant association between the DASH diet and the risk of HTN over a 6.3-year follow-up duration [8].

Recently, a novel dietary pattern known as the MED-DASH Intervention for Neurodegenerative Delay (MIND) has been created, combining the MED and DASH diets [9]. This diet has been hypothesized to be effective in age-related neurodegenerative disorders [10]. Compared with the two previous dietary indices, the MIND diet not only includes cheese, fast/fried foods, and butter/margarine as separate food groups, but also its major focus is on the consumption of berries and green leafy vegetables rather than considering all types of fruits or vegetables as a general category. In addition, the MIND diet suggests olive oil as the primary oil for daily consumption [9]. The association between the MIND diet and blood pressure has been investigated only in a cross-sectional study to the best of our knowledge. The findings of the study did not show any significant association between the MIND diet and odds of high blood pressure [11].

Due to the continuous increase in the prevalence of HTN, and the necessity of investigating the association between different dietary indices and HTN in different populations, we aimed to examine the prospective associations between the adherence to the MED, DASH, and MIND diets and the incidence of HTN in a cohort of Iranian adults.

## Methods

### Study population

**Fig. 1:**
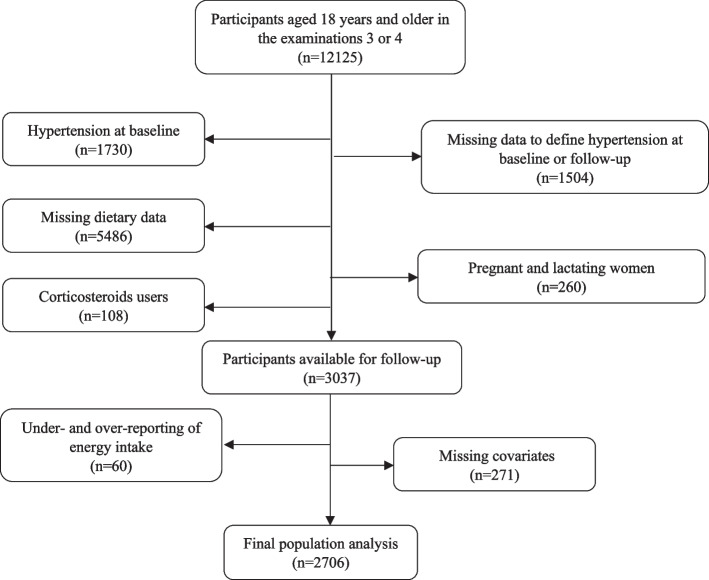
Selection of the study sample

The Tehran Lipid and Glucose Study (TLGS) is a continuing prospective community-based study initiated in 1990–2001 by enrolling 15005 individuals between the ages 3 to 69 years from Tehran's district 13 [12]. Following the initial examinations, participants were followed-up every three years to update their information. Dietary data from examination 3 (2005–2008) were gathered using a food frequency questionnaire (FFQ). Of the 12125 people aged ≥ 18 years in the examinations 3 or 4 (2008–2011), which we consider as the baseline phases for our present analysis, those with missing information at baseline or follow-up to define HTN status (*n* = 1504), those with prevalent HTN (*n* = 1730), pregnant and lactating women (*n* = 260), corticosteroid users (*n* = 108), and those with missing information on dietary assessment (*n* = 5487) were excluded. We further excluded individuals with implausible energy consumption according to the sex-specific 1st and 99th percentiles of energy intakes (*n* = 60) and those who were missing covariates (*n* = 271). Finally, 2706 individuals were tracked till the end of examination 6 (2014–2018) for a median follow-up of 7.4 years (Fig. 1). In TLGS, a subsample of participants was chosen at random to complete dietary information. Comparisons of the characteristics of participants with and without dietary data in either the third or fourth examination showed that the proportion of males, smokers, those with a family history of cardiovascular disease, those with an academic education, and the level of physical activity were similar between the two groups. However, the age and body mass index (BMI) of individuals who completed the FFQ in the fourth examination were slightly lower than those without dietary data (40.8 ± 14.1 vs. 44.8 ± 17.1 years and 27.3 ± 4.9 vs. 27.7 ± 5.2 kg/m^2^, respectively) [8, 13]. All methods of the present study has been carried out in accordance with the Declaration of Helsinki. The present study was approved by the ethics committee of the Research Institute for Endocrine Sciences, Shahid Beheshti University of Medical Sciences (IR.SBMU.ENDOCRINE.REC.1400.098). A written informed consent form was obtained from every individual (Fig. 1).

### Demographic, anthropometric, and physical activity assessments

Data regarding age, sex, educational level, smoking status, medical history, and medication use were collected by a questionnaire. We classified individuals into two groups for education: ≤12 years or > 12 years (academic education) and three groups for smoking status: nonsmokers, ex-smokers, and current smokers. Any cardiovascular disease incidents in first-grade female family members 65 years or younger or first-grade male family members 55 years or younger were regarded to have a premature family history of cardiovascular disease [14].

Weight was measured with light clothing to the nearest 0.1 kg using a digital weighing scale (Seca 707; Seca Corporation, Hanover, Maryland; range, 0.1–150 kg). Height was assessed in a standing position using a stadiometer to the nearest 0.1 cm, while shoes were removed and shoulders were in normal alignment. Body mass index (BMI) was calculated by dividing weight (Kg) by height squared (m^2^).

Physical activity over the past year was assessed using the Modifiable Activity Questionnaire (MAQ) [15]. The MAQ consists of two types of questions based on leisure activities and work-related activities. Participants reported the frequency and time spent on every activity in each category based on four types of intensity (light, moderate, hard, and very hard). The physical activity was expressed as metabolic equivalent minutes per week (MET-min/wk).

### Biochemical measurements

After an approximate 12-to-14-hour overnight fast, blood samples of all participants were collected in a sitting position between 7:00 and 9.00 AM. Samples were immediately centrifuged within 30–45 min. Fasting serum glucose (FSG), total cholesterol (TC), and triglycerides (TGs) were assessed using an enzymatic colorimetric method. Serum high-density lipoprotein-cholesterol (HDL-C) was assessed after precipitation of the apolipoprotein B-containing lipoproteins with phosphotungstic acid. Inter/intra-assay coefficient variations (CVs) were both 2.2% for FSG, 0.5 and 2% for TC and HDL-C, and 0.6 and 1.6% for TGs, respectively) [16]. Serum creatinine levels were measured using kinetic colorimetric Jaffe with a sensitivity of 0.2 mg/dL (range, 18–1330 µmol/L (0.2–15 mg/dL) and both intra- and inter-assay CVs were less than 3.1%. All blood samples were analyzed at the TLGS research laboratory on collection using the Selectra 2 auto-analyzer (Vital Scientific, Spankeren, Netherlands). Biochemical measurements were performed using commercial kits (Pars Azmoon Inc., Tehran, Iran).

### Dietary assessments

Participants' dietary intakes were examined by trained dietitians using the semi-quantitative FFQ.

The FFQ's validity and reliability have already been assessed [17]. Participants were asked to estimate their consumption of each food item during the preceding year using daily, weekly, or monthly frequency and predetermined portion sizes. Using household measurements, all ingested food portion amounts were converted to grams. The absolute dietary component consumption were used to estimate each dietary score. A higher score for any of the dietary indices suggests more adherence.

The MED score was computed using the approach published by Trichopoulou et al [18]. The method includes 9 components: vegetables, legumes, fruits and nuts, cereals, fish, meat and meat products, dairy products, the ratio of monounsaturated fatty acids (MUFA) to saturated fatty acids (SFA), and alcohol. We did not consider alcohol intake as a food component due to lack of information. Values of 0 or 1 were assigned to each component, using sex-specific median as cut-off. Thus, for the 5 expected beneficial components (vegetables, legumes, fruits and nuts, cereals, and fish), individuals whose consumption was at or above the sex-specific median were assigned a value of 1, and a value of zero was assigned for individuals whose consumption was below the median. Similarly, a value of 1 was assigned if the MUFA to SFA ratio was equal or above the sex specific median value and a value of zero was assigned for consumption below the median. For components presumed to be detrimental (meats or meat products and dairy products), 1 point was assigned if consumption was below the sex-specific median value and participants whose consumption was at or above the median were assigned a value of 0. Finally, the total MED scores ranged from zero to 8.

The DASH score was calculated by Epstein et al. based on the intakes of 10 food components: total grain, fruits, vegetables, nuts, seeds and dry beans, dairy, meats, poultry, and fish, %energy intake from total fat, % energy intake from saturated fat, sweets, and sodium. Based on the recommended number of servings, a score of 1, 0.5, or 0 was assigned to each of the 10 dietary components, and the scores were then summed [19]. The DASH score ranged between 0 and 10.

The MIND score was developed by Morris et al. [9]. Since our FFQ did not inquire about main oil and alcohol consumption, these dietary components were omitted from our MIND score computation. Therefore, the following 13 food groups were considered to calculate MIND score: whole grains, green leafy vegetables, other vegetables, berries, red meats and products, fish, poultry, beans, nuts, fast or fried foods, butter, margarine, pastries or sweets. According to the recommended number of servings, each component was assigned a score of 1, 0.5, or 0; these scores were then combined. The MIND score ranged between 0 and 13.

### Blood pressure assessment and disease definitions

Blood pressure was measured by a trained physician in a seated position twice with a 1-minute interval on the right arm using a mercury sphygmomanometer. Before the assessment, every participant was asked to rest for 15 min while sitting. The average of the two measurements was considered as the final blood pressure measure.

In the absence of antihypertensive medication, prehypertension was defined as having an SBP of 120–139 mmHg and/or a DBP of 80–89 mmHg [20]. HTN was defined based on the seventh report of the Joint National Committee on prevention, detection, evaluation, and treatment of high blood pressure (JNC-VII) [1] as systolic blood pressure (SBP) ≥ 140 mmHg or diastolic blood pressure (DBP) ≥ 90 mmHg, or the use of blood pressure-lowering agents. A pre-tested questionnaire was used to assess the use of any blood pressure-lowering agents, including diuretics, beta-blockers, angiotensin-converting enzyme inhibitors, calcium channel blockers, and angiotensin receptor blockers at baseline and throughout the follow-up visits.

Type-2 diabetes mellitus (T2DM) was defined as FSG ≥ 126 mg/dl or 2-h post-challenge plasma glucose ≥ 200 mg/dl, or taking anti-diabetic medications [21]. Chronic kidney disease (CKD) was defined based on the calculation of estimated glomerular filtration rate (eGFR) using the CKD Epidemiology Collaboration (CKD-EPI) equation [22]. Dyslipidemia was also described as having one or more of the following criteria: serum TGs of ≥ 200 mg/dL, TC of ≥ 240 mg/dL, HDL-C < 40 mg/dL, or taking any lipid-lowering medications [23].

### Statistical analysis

We examined histogram charts to evaluate if the variable distribution was approximately normal. Baseline characteristics of the participants across quartile categories of each dietary score were compared using analysis of variance (ANOVA) for continuous variables and chi-square test for categorical variables. Before analysis, non-normally distributed variables such as physical activity, FSG, TGs, and caffeine and olive intakes were natural log transformed. Data were presented as Mean ± standard deviation (SD) for normally distributed variables, median (interquartile range) for skewed variables, and percent for categorical variables. Associations between the dietary indices and the risk of HTN were examined using Cox proportional hazards regression analysis. Hazard ratio (HR) and 95% confidence interval (CI) was estimated per one unit change in each dietary score (as a continuous variable) and across their quartile categories, considering the first group as a reference. The associations were adjusted for age (continuous) and sex in the model 1, and additional adjusted for physical activity (continuous), academic education (yes/no), premature family history of cardiovascular disease (yes/no), smoking (smokers, non-smokers, and ex-smokers), baseline BMI, baseline prevalence of disease including T2DM (yes/no), CKD (yes/no), prehypertension (yes/no), and dyslipidemia (yes/no), aspirin intake (yes/no), and dietary intakes of total energy (continuous), and olive oil (continuous). The median value was assigned to each quartile and treated as a continuous variable to compute *P* for trend. To investigate the interactions between sex and BMI with dietary scores for the risk of HTN, interaction terms were included in multivariable Cox model. The proportional hazard assumption of the multivariable Cox model was assessed using Schoenfeld’s global test of residuals.

The event time of HTN was interval-censored because the precise onset time was uncertain, despite the probability that the event occurred between the two examination appointments. For the analysis of the interval-censored outcome, therefore, midpoint censoring was utilized. According to mid-point censoring, the event date of HTN was defined as the midpoint between the data of the follow-up examination when HTN was first identified and the most recent follow-up examination previous to diagnosis. The follow-up time was also calculated based on the difference between the estimated mid-time date and the date at which the individuals entered the study. The survival time was the interval between the first and the last examination dates for the censored individuals.

Several sensitivity analyses were performed, removing individuals with 1-early diagnosis of HTN (less than 2 years), 2-late diagnosis of HTN (more than 10 years), and 3-prehypertension at baseline, 4-T2DM at baseline, and 5-CKD at baseline. All analyses were performed using IBM SPSS for Windows version 20 (IBM, New York, USA) with a two-tailed *P* value < 0.05 being considered significant.

## Results

The mean ± SD for age and BMI of the participants were 37.9 ± 12.5 years and 26.7 ± 4.72 kg/m^2^, respectively. Women made up 52.4% of the total. The mean (range) of MED, DASH, and MIND score in the participants were 4.4 (0, 8), 4.8 (1–9), and 6.8 (2.5–11.5), respectively. Table 1 shows the baseline characteristics of the participants according to the quartiles of these dietary scores. Participants in the upper quartiles of the DASH and MIND diets compared to the lower quartiles were older, more physically active, had higher baseline FSG and SBP, and were more likely to use aspirin. Those in the highest quartile of the DASH and MIND scores had greater HDL-C, TGs, and DBP values than those in the first quartile. Furthermore, the prevalence of prehypertension, T2DM, and dyslipidemia was greater in quartile 4 of the DASH diet compared to quartile 1. Energy and olive oil consumption were significantly higher in the highest quartiles of all three dietary scores compared to the lowest quartiles.

**Table 1:** Baseline characteristics of participants according to the quartiles of dietary indices ^a^

Variables	Total	MED			DASH			MIND		
		Q1 (*n* = 733)	Q4 (*n* = 224)	*P*-value ^b^	Q1 (*n* = 922)	Q4 (*n* = 654)	*P*-value ^b^	Q1 (*n* = 858)	Q4 (*n* = 461)	*P*-value ^b^
**Demographic variables**										
Age, years	37.9 ± 12.5	38.3 ± 12.5	38.2 ± 12.6	0.022	36.1 ± 11.7	39.9 ± 13.3	< 0.001	35.1 ± 11.1	42.7 ± 13.9	< 0.001
Sex, female, %	52.4	52.8	52.2	0.894	59.3	52.4	< 0.001	53.4	51.8	0.890
Body mass index, kg/m^2^	26.7 ± 4.72	26.6 ± 4.70	27.1 ± 4.90	0.334	26.2 ± 4.60	27.4 ± 4.80	< 0.001	26.0 ± 4.45	27.3 ± 4.70	< 0.001
Physical activity, Met-min/week	1619(4359)	1429(3596)	1727(5262)	0.106	1340(3058)	2117(5364)	< 0.001	1121(3043)	2501(5463)	< 0.001
Academic education, %	27.8	27.7	26.3	0.665	26.6	29.7	0.121	25	32.3	0.018
Smoking, %										
* Current smoker*	14.2	14.9	13.4	0.583	14.8	14.4	0.048	14.9	13.2	0.436
* Non-smoker*	77	76.4	76.3		78.2	75.2		77.6	77.6	
* Ex-smoker*	8.8	8.7	10.3		7.0	10.4		7.5	9.1	
**Biochemical & blood pressure variables**										
Fasting serum glucose, mg/dl	87(12)	87.0(12.0)	88.0(12.8)	0.062	86(11.0)	89(13.0)	< 0.001	86 (11.0)	89 (13.0)	< 0.001
Total cholesterol, mg/dl	184 ± 38.2	182 ± 37.6	182 ± 36.2	0.021	182 ± 37.3	185 ± 37.5	0.236	181 ± 37.6	186 ± 38.6	0.036
High-density lipoprotein cholesterol, mg/dl	43.5 ± 1.70	43.6 ± 10.7	43.4 ± 10.1	0.953	43.9 ± 10.8	44.1 ± 11.0	0.042	43.5 ± 10.8	44.8 ± 11.4	0.030
Triglyceride, mg/dl	115(107)	111(78.0)	114(86.5)	0.160	109(83.3)	120(91.0)	0.007	113(85)	115(83)	0.013
Systolic blood pressure, mmHg	109 ± 12.2	108 ± 13.0	109 ± 11.6	0.038	107 ± 12.4	110 ± 12.5	< 0.001	107 ± 12.1	111 ± 12.7	< 0.001
Diastolic blood pressure, mmHg	72.1 ± 8.75	71.5 ± 8.90	72.5 ± 8.0	0.231	71.2 ± 8.95	73.1 ± 8.50	< 0.001	71.3 ± 9.10	72.5 ± 8.70	0.008
**Disease prevalent**										
Prehypertension, %	33.0	30.7	33.0	0.407	29.0	40.5	< 0.001	30.3	35.6	0.126
Diabetes, %	4.7	4.0	4.5	0.711	3.1	7.0	< 0.001	3.5	5.7	0.169
Chronic kidney disease, %	6.8	6.8	7.6	0.394	6.6	6.1	0.238	7.4	7.9	0.238
Dyslipidemia, %	48.4	45.3	44.6	0.095	44.1	50.9	0.016	47.5	47.6	0.500
Premature family history of CVD, %	9	9.8	8.9	0.784	8.9	7.6	0.373	10.7	7.1	0.071
Aspirin medication, %	5.8	4.8	4.9	0.338	3.7	8.6	0.001	3.9	9.0	< 0.001
**Dietary intakes**										
Energy, kcal/d	2448 ± 929	1974 ± 676	2932 ± 929	< 0.001	2077 ± 734	2811 ± 1018	< 0.001	2259 ± 847	2653 ± 930	< 0.001
Carbohydrate, % of energy	54.9 ± 7.24	54.4 ± 6.82	61.8 ± 6.48	< 0.001	53.7 ± 6.23	63.0 ± 5.87	< 0.001	56.2 ± 7.47	59.1 ± 6.94	< 0.001
Protein, % of energy	13.9 ± 2.63	14.2 ± 2.79	13.1 ± 2.21	< 0.001	13.6 ± 3.02	14.4 ± 2.31	< 0.001	13.1 ± 2.57	15.0 ± 2.54	< 0.001
Fat, % of energy	31.2 ± 6.97	33.7 ± 6.90	28.3 ± 6.77	< 0.001	34.9 ± 6.11	26.1 ± 5.33	< 0.001	32.9 ± 7.44	29.4 ± 6.16	< 0.001
Caffeine, g/d	105(86)	104(105)	106(106)	0.260	104(101)	105(118)	0.431	104(103)	105(118)	0.935
Olive & Olive oil, g/d	0.68(2.56)	0.54(1.82)	0.87(2.81)	< 0.001	0.47(1.53)	0.92(3.51)	< 0.001	0.35(1.29)	1.33(4.56)	< 0.001

*CVD* Cardiovascular disease, *DASH* Dietary Approaches to Stop Hypertension, *MED* Mediterranean diet, *MIND* Mediterranean-DASH Intervention for Neurodegenerative Delay

^a^ Data are presented as mean ± SD for normally-distributed quantitative variables, median (interquartile range) for non-normally distributed quantitative variables, and % for categorical variables

^b^ Based on analysis of variance or chi-square tests, as appropriate

During a median follow-up of 7.4 years (quartile 1, quartile 3: 4.6, 9.0 years), 18262 person-years follow-up, 599 incident HTN were identified (294 men and 305 women). Table 2 shows the associations between dietary scores and the incidence of HTN. There was no significant relationship between the dietary scores and the risk of HTN, either as continuous or categorical variables. Results were not changed after excluding individuals with early or late diagnosis of HTN, those with prehypertension, T2DM, or CKD at baseline (data not shown).

**Table 2:** Hazard ratio (95% confidence interval) for hypertension based on dietary indices

Dietary indices	Continuous	*P*-value	Quartile of dietary scores				*P*-trend
			Q 1	Q 2	Q 3	Q 4	
**MED**							
Mean score (minimum, maximum )	4.4 (0, 8)		2.5 (0, 3)	4 (4)	5.4 (5,6)	7.1 (7, 8)	-
Number of HTN events	599		167	155	235	42	
Person-years	18262		5034	4537	7200	1491	
Incidence (per 1000 person years)	32.8		33.2	34.2	32.6	28.2	
Age- and sex-adjusted	1.00 (0.95, 1.05)	0.904	1.00	1.04 (0.84, 1.30)	1.06 (0.87, 1.29)	0.83 (0.59, 1.16)	0.739
Multivariable-adjusted	0.98 (0.93, 1.04)	0.577	1.00	0.96 (0.77, 1.20)	0.99 (0.80, 1.23)	0.76 (0.53, 1.09)	0.388
**DASH**							
Mean score (minimum, maximum )	4.8 (1–9)		3.5 (1–4)	4.7 (4.5-5)	5.5 (5.5 )	6.5 (6–9)	
Number of HTN events	599		182	177	80	160	
Person-years	18262		6582	5440	2163	4077	
Incidence (per 1000 person years )	32.8		27.7	32.5	37.0	39.0	
Age- and sex-adjusted	1.03 (0.97, 1.10)	0.386	1.00	1.04 (0.84, 1.28)	1.19 (0.91, 1.54)	1.14 (0.92, 1.41)	0.168
Multivariable-adjusted	0.99 (0.92, 1.05)	0.661	1.00	0.95 (0.76, 1.17)	1.15 (0.87, 1.50)	0.99 (0.79, 1.25)	0.771
**MIND**							
Mean score (minimum, maximum )	6.8 (2.5–11.5)		5.4 (2.5-6)	6.8 (6.5-7)	7.5 (7.5)	8.6 (8-11.5)	
Number of HTN events	599		134	161	184	120	
Person-years	18262		6348	4078	5005	2830	
Incidence (per 1000 person years)	32.8		21.1	39.5	36.8	42.4	
Age- and sex-adjusted	1.02 (0.96, 1.09)	0.445	1.00	1.13 (0.92, 1.39)	1.38 (1.06, 1.79)	1.09 (0.87, 1.35)	0.238
Multivariable-adjusted	1.01 (0.95, 1.08)	0.662	1.00	1.14 (0.93, 1.41)	1.27 (0.97, 1.65)	1.08 (0.86, 1.36)	0.335

Multivariable models adjusted for age (continuous), sex, physical activity (continuous), academic education (yes/no), premature family history of CVD (yes/no), smoking (smoker/non-smoker/ex-smoker), baseline BMI (continuous), baseline prevalent of diseases including chronic kidney disease (yes/no), diabetes mellitus (yes/no), pre-hypertension (yes/no) and dyslipidemia (yes/no), aspirin intake (yes/no) and dietary total energy (continuous), caffeine (continuous), and olive intake (continuous)

*HTN* Hypertension, *DASH* Dietary Approaches to Stop Hypertension, *MED* Mediterranean diet, *MIND* Mediterranean-DASH Intervention for Neurodegenerative Delay

**Fig. 2:**
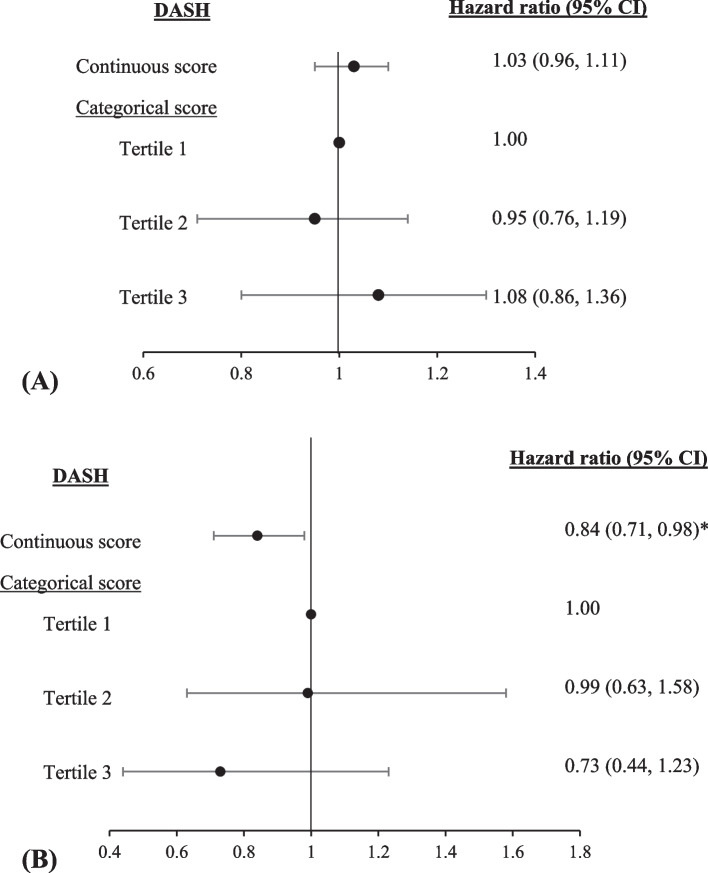
Hazard ratio (95% CI) for hypertension according to dietary indices separately in overweight/obese individuals (**A**, *n* = 1677) and normal-weight (**B**, *n* = 1029). Adjusted for age (continuous), sex, physical activity (continuous), academic education (yes/no), family history of CVD (yes/no), smoking (smoker/non-smoker/ex-smoker), baseline BMI (continuous), baseline prevalent of diseases including CKD(yes/no), diabetes mellitus (yes/no), pre-hypertension (yes/no) and dyslipidemia (yes/no), aspirin intake (yes/no) and dietary total energy (continuous), caffeine (continuous), and olive intake (continuous). *P = 0.031

Significant interactions were found between BMI and DASH (*P*-interaction < 0.001). Therefore, we conducted stratified analyses based on baseline BMI status [normal-weight (BMI < 24.9 kg/m^2^) and overweight (BMI ≥ 24.9 kg/m^2^)] for DASH score. Stratified analysis based on BMI status revealed a significant inverse association between DASH diet as a continuous variable and the risk of HTN in normal-weight adults (HR = 0.84, 95%CI = 0.71–0.898, *P* = 0.031) in the multivariable-adjusted model (Fig. 2B). This finding was not seen in adults with overweight/ obesity (Fig. 2A).

## Discussion

In this prospective study, we could not find any significant associations between the MED, DASH, or MIND dietary scores and the incidence of HTN among Iranian adults. However, as a continuous variable, the DASH diet was associated with a lower risk of HTN in normal-weight participants after adjustment for all potential covariates.

Although several meta-analyses of RCTs demonstrated the beneficial effects of MED and DASH diets on the treatment of HTN [4, 5], there is a scarcity of observational data and inconsistency about the association between MED and DASH as a priori diet scores and the prevention of HTN. Most of the previous observational studies that evaluated this association were cross-sectional [7, 11, 24, 25], and there are limited longitudinal prospective investigations in this regard [26–30].

Similar to the current findings, a prospective study conducted among a Mediterranean population (the SUN cohort) could not find any beneficial association between adherence to the MED diet and the risk of HTN in 9408 Spanish men and women after a median follow-up of 4.2 years [26]. The other investigation conducted among this cohort with a longer follow-up duration and a higher number of participants suggested that more adherence to the MED diet in combination with other healthy lifestyle factors, such as non-smoking, physical activity, a low BMI, moderate alcohol consumption, and the avoidance of binge drinking, was associated with a decreased risk of HTN over a median follow-up of 10 years. However, when the individual contribution of the MED diet was investigated, the inverse association between the MED diet and risk of HTN was marginally significant [HR (95% CI) = 0.82 (0.79-1.00) in those with a MED score ≥ 4 compared to those < 4] [27]. The SUN cohort studies used the 9-point Trichopoulou et al. scoring method to evaluate MED diets similar to ours, but participants' self-reports were used to figure out the incidence of HTN. In contrast, a cohort of middle-aged Australian women demonstrated an inverse association between MED score and the odds of self-reported HTN after 15 years of follow-up [28]. The study generated the MED diet score according to Sofi et al., a scoring system in which the cut-off values were specified for each food component to more accurately represent the health-promoting potential of the traditional MED diet [31]. In our study, only 8.3% of the participants had the highest adherence to the MED diet (highest quartile), while 35% of the population in the Australian study was within the highest quartile of the MED diet. Consequently, the level of adherence to the MED diet among the majority of our study population may not have been high enough to identify any favorable association between this diet and blood pressure. However, in a recently published meta-analysis, there was a high heterogeneity across the findings of observational studies investigating the association between MED, SBP, and DBP. This meta-analysis by pooling data of 54 studies showed a slightly lower SBP in those with the highest vs. lowest adherence to the MED diet, but DBP did not differ significantly between the two groups. In the majority of the included studies in this meta-analysis, which had a cross-sectional design, the mean SBP of groups with high and low adherence to MED was normal (SBP = 130 mmHg) [32]. Therefore, the MED diet alone may not be sufficient to prevent the incidence of HTN.

Regarding the DASH score, a prospective study of 20993 Caucasian women indicated no significant association between more adherence to the DASH and the risk of self-reported HTN over 11 years of follow-up after accounting for other risk factors. As no women in this investigation achieved total adherence to the DASH diet and few women (19% with a score of 6.5–10) obtained high adherence, the authors concluded that very high concordance, as observed in the DASH trials, might be necessary to see the benefits of the DASH diet [29]. A prospective study among Chinese adults also showed that the participants may not benefit from DASH unless they rigorously adhere to the diet recommendation. This study investigated the association of DASH diet alone or in combination to normal BMI and moderate or heavy physical activity with the risk of HTN. Interestingly, they found that having a normal BMI in combination with adopting the highest concordance to DASH was related to a 34% (95% CI = 20–46%) lower risk of HTN. Adopting all three components of a low-risk was related to a 42% lower risk of HTN (95% CI = 29–53%) [30]. In the current study, we observed the inverse association between DASH and risk of HTN in normal-weight individuals, which is in line with previous studies suggesting a combination of healthy lifestyle factors such as having a normal BMI along with a healthy diet may be important for the prevention of HTN [27, 30].

The initial development of the MIND diet was based on available evidence relating dietary components to cognitive decline prevention. Despite the general similarity to the MED and DASH diets, the MIND diet specifies consuming olive oil as the primary oil, green leafy vegetables, cheese (but not other dairy products), berries (but not other fruits), and nuts as separate food groups. In addition, fast foods, fried foods, butter, and margarine consumption are also considered in the MIND diet that are not included in the computation of MED and DASH scores [9]. Recently, investigating the association between MIND and cardio-metabolic health is of interest. A cross-sectional study conducted among 836 middle-aged Iranian adults could not find any significant association between the MIND and the elevated BP as a component of metabolic syndrome [11]. In the present prospective study in which we evaluated the association between the MIND diet and the risk of HTN for the first time, we were unable to find any significant findings.

The study's strengths include its prospective design, evaluation of dietary intakes using a valid FFQ, computation of three distinct a priori dietary scores, and determination of the incidence of HTN based on objective measurements of SBP and DBP, as well as consideration of BP-lowering medications. Different sensitivity analyses were conducted to account for variables that could alter the results, which is another merit of this study.

The limitations of the present work should also be noted. First, despite using the valid FFQ, measurement errors in assessing food intakes are possible due to the recall bias and reporting bias of the participants [33]. Second, the dietary scores were calculated using dietary data collected at baseline, which may not reflect the participants' actual dietary intakes throughout the study. Third, we lack information regarding alcohol consumption, which is one of the components of the MED and MIND scores. In addition, primary olive oil use was removed from the MIND computation due to a lack of data. As a result, we could not provide a comprehensive feature of diet adherence based on their original scoring system. Fourth, the participants were selected from Tehran's district 13, which may not be representative of the Iranian population. Consequently, the generalizability of our findings is restricted to this group of participants.

## Conclusions

The present study showed that none of the MED, DASH, or MIND diets were associated with the reduced risk of HTN in a cohort of Iranian adults. However, higher DASH scores (as continuous variables) in normal-weight adults were associated with a lower risk of HTN. More prospective study is necessary to assess whether adherence to the MED, DASH, or MIND diets alone or in combination with other healthy lifestyle characteristics is an effective strategy for preventing HTN.

## Data Availability

All the datasets are available from the corresponding author on reasonable request.

## Abbreviations

DASH: Dietary approach to stop hypertension
MIND: Mediterranean-DASH Intervention for Neurodegenerative Delay
MED: Mediterranean
HTN: Hypertension
CKD: Chronic kidney disease
HR: Hazard ratio
RCT: Randomized controlled trial
FFQ: Food frequency questionnaire
BMI: Body mass index
MAQ: Modifiable activity questionnaire
MET: Metabolic equivalent
FSG: Fasting serum glucose
TC: Total cholesterol
TG: Triglycerides
HDL-C: High-density lipoprotein-cholesterol
CV: Coefficient variations
LDL-C: Low-density lipoprotein cholesterol
JNC-VII: Joint national committee on prevention, detection, evaluation, and treatment of high blood pressure
SBP: Systolic blood pressure
DBP: Diastolic blood pressure
T2DM: Type-2 diabetes mellitus
eGFR: Estimated glomerular filtration rate
CKD-EPI: CKD Epidemiology Collaboration
ANOVA: Analysis of variance
SD: Standard deviation
